# Exploring reddit forum for software evolution as an alternative requirements source: An end-user discussion dataset on Google maps

**DOI:** 10.1016/j.dib.2024.110993

**Published:** 2024-10-03

**Authors:** Javed Ali Khan, Nek Dil Khan, Muhammad Yaqoob, Affan Yasin, Ayed Alwadain

**Affiliations:** a Department of Computer Science, School of Physics, Engineering, and Computer Science, University of Hertfordshire, Hatfield AL10 9AB, UK; b Faculty of Information Technology, Beijing University of Technology, Beijing 100124, China; c School of Software, Northwestern Polytechnical University, Xian 710072, Shaanxi, China; d Computer Science Department, Community College, King Saud University, Riyadh 11421, Saudi Arabia

**Keywords:** Requirements engineering, CrowdRE, Reddit discussions, Requirements dataset, Google maps feedback, Sentiment analysis, Classification, Natural Language Processing (NLP)

## Abstract

For software development and evolution, end-user feedback from app stores and the Twitter (X) platform has been intensively used recently. However, Reddit forums that provide an argumentative platform to argue and reason about various software features and issues have been less likely to be explored for software evolution and improvement in the literature. Therefore, this study explores Reddit forums as an alternative source for software evolution compared to App Stores, Twitter (X), and Amazon reviews. For this purpose, a Python script is developed to extract end-user discussions related to the Google Maps (GM) app from Reddit forums using Python Praw API, keep the original argumentative structure in user discussions. In total, 3119 end-user discussions from seven related topics about the GMM app are extracted for software evolution. This dataset includes detailed end-user feedback and associated metadata, including Comments ID, Parent ID, author names, timestamps, and upvotes. This dataset is a crucial and valuable resource for software vendors, developers, researchers, and educationists to improve their understanding of identifying new features to include in upcoming app versions. Also, it is of pivotal importance in better understanding recently occurring issues, unlike app stores where user debate on it and provide their justifications. Moreover, the replication package and process of the dataset can enable software researchers, vendors, and developers to extract data from the Reddit forum and use it for the software evolution and improvement process.

## Specifications Table

For enhanced clarity, the data specifications are presented in Table 1.

**Table 1:** Specifications Table.

Subject	Software Engineering, Software Education, and Computer Science.
Specific subject area	Software evolution, Crowd Requirements Engineering, and Mining software repositories.
Type of data	Tabular.
Data collection	A Python script is developed to extract user discussions about the Google Maps (GM) app from the Reddit forum using the Python PRAW library).
Data source location	University of Hertfordshire, College Lane, Hatfield, Hertfordshire, AL10 9AB, United Kingdom.
Data accessibility	The dataset is published, whose details are provided below Repository name: Mendeley Data identification number: 10.17632/kng6zs9t5y.1 Direct URL to data: https://data.mendeley.com/datasets/kng6zs9t5y/1 GitHub Link: https://github.com/nekdil566/dataset/blob/main/README.md Instructions for accessing these data: To access the dataset, copy the URL and paste it to any browser
Related research article	J.A. Khan, A. Yasin, R. Fatima, D. Vasan, A.A. Khan, A.W. Khan, Valuating requirements arguments in the online user's forum for requirements decision-making: the CrowdRE-Varg framework. Software: Practice and Experience. John Wiley & Sons Ltd; 2022. https://onlinelibrary.wiley.com/doi/abs/10.1002/spe.3137

## 1 Value of Data

• *Proposed Dataset Use Case*: 3119 end-user comments from the Reddit forum across seven related discussion topics on the Google Map (GM) app have been extracted to provide software vendors and researchers opportunities to listen to alternative voices about software evolution. The dataset was developed to identify conflict-free new features and issues using the theory of argumentation (CrowdRE-Arg), i.e., bipolar argumentation and coalition-based argumentation, by analyzing supporting and attacking arguments for each new feature and issue [3]. Also, the dataset is utilized to identify rational information from the Reddit forum and identify key stakeholders who frequently contribute rationale information [1]. Additionally, the CrowdRE-Arg approach is further improved by proposing a valuation-based CrowdRE-approach (CrowdRE-VArg) that identifies conflict-free new features and issues by valuating their supporting and attacking arguments using valuation-based argumentation [2]. Moreover, the dataset could be a valuable source for researchers, vendors, and developers to understand the hidden patterns and recover valuable information about software improvement and evolution, such as.
• *Alternative Requirements Engineering Source to Understand Rationale on Requirements decisions*: The dataset can be a potential alternative source for gathering end-user feedback, opinions, and sentiments about frequently proposing new features, issues occurring, or user experience with the software applications compared to app stores and Twitter (X) social media platforms. Also, the end-user discussions on the Reddit forum are more detailed than other datasets collected from app stores, Twitter (X), etc., allowing users to elaborate on the issues they confronted or provide a rationale for demands for a new feature. Moreover, the Reddit forum provides an argumentative structure of reply comments to an n-level, resulting in rich software-related information that researchers and vendors can utilize for informed decision-making on giving a rationale for why a feature or issue is selected for the coming version.
• *Rich Source of Active Stakeholders and Feature/Issues Prioritization*: The dataset allows software developers to identify key stakeholders and gain a bird's eye view of their frequently contributed requirements-related information on Reddit forums. On Reddit, there are 267.5 million weekly active users, and 2.7 million have joined the Andriod subreddits relevant to software development and evolution, where registered users frequently post information about user experiences, issues, or new features required for the software applications. Therefore, researchers and vendors can develop approaches that identify and listen to the stakeholders contributing valuable information in the Reddit forum to improve user satisfaction [1]. Furthermore, software developers can use the dataset to propose rationale-based requirements prioritization approaches. They can harness the rationale to negotiate the priorities of frequently reported new features or issues to be included in the next release of the software application. The dataset can be used as a case study to validate the rationale-based prioritization approaches.
• *Training and validating Machine/Deep and Transfer Learning Algorithms*: The dataset can serve as a baseline source to train and validate various machine/deep and transfer learning classifiers in automatically identifying and classifying requirements-related information from Reddit forums. The dataset is an alternative source for these classification algorithms to determine their capabilities and performance in classifying requirements-related information on comparatively larger end-user feedback than short user comments from app stores, Twitter (X), etc. It would help recover challenges associated with the classification approaches in correctly identifying and classifying requirements-related information when introduced to more sparse end-user comments [2,3]. Moreover, the dataset can be used to cross-validate the performance of classifiers trained on comparatively short text user feedback with a similar nature, i.e., the dataset used by Kurtanovic and Maalaj [4].
• *Argumentation Mining*: The dataset can be used by social science, computer science, and software engineering researchers to make informed decisions using argumentation theory [5] and advanced NLP approaches. For example, argumentation mining can automatically be employed in the dataset to identify argumentative-related information, such as assumption or conclusion, premises, or the complete argument [6]. Also, the dataset can be utilized to determine the relationship between the sentences, i.e., supporting, attacking, or natural, that would help make informed decisions underneath argumentation theory. Another challenge for software vendors is that users submit feedback on social-media platforms that need summarising for informed decision-making using weighted [7] and bipolar [8] abstract argumentation. For this purpose, the dataset fits best as it poses an argumentative structure; researchers can utilise it for this purpose. Furthermore, the dataset can be used to compare different apps (Google Maps, CityMapper, Waze, etc.) to identify the app's performances and limitations to the most frequently mentioned one in the end-user feedback.
• *Educational Resource for Automated Software Engineering*: The automated software engineering discipline has recently been embarked on in academia to portray emerging trends and concepts in mining software repositories to students for software evolution and continuous improvements. For this purpose, the dataset can be a practical source for academic students and educators to teach automated software engineering, data science, NLP, and social media analytics. Various machine learning training and validation concepts, such as preprocessing, feature engineering, data balancing, and various evaluation matrices, including accuracy, precision, recall, f-measures, and confusion matrics, can be demonstrated with the dataset in the classroom and labs due to its easy availability and different nature compared to existing datasets. The end-user conversation is quite lengthy compared to the Play Store datasets.
• *Redesign Existing Discussion Forums*: The dataset can serve as a validation source for software researchers and vendors in extending the functionalities of the current social media platforms such as Reddit forum, etc., by providing certain statistical operations over the end-user feedback, i.e., providing an overview of the most frequently discussed features or issues to provide opportunities for the new users to get involved in the ongoing discussion which is currently hard to skim through the whole discussion. Moreover, the dataset can serve as a baseline source for researchers to identify frequently occurring important topics and trends using supervised learning algorithms [9], i.e., topic modelling, clustering, etc., and show overall directions to the incoming users on the discussion to give them an initial quick overview of the ongoing discussion. The detailed data specification is elaborated in Table 1 for improved clarity for the research community and software vendors.

## 2 Background

The creation of this dataset was driven by the increasing recognition of the significance of understanding online discussions and user behaviors on the internet site known as Reddit. Understanding end-user engagement, emotions, and discussion topics is crucial for many groups involved in the various informal landscapes of the internet, including social media platforms, content creators, and researchers. The focus on analyzing comments from a particular thread demonstrates a methodological approach that prioritizes accuracy, enabling a complete exploration of online discussions. The dataset offers a comprehensive analysis of end-user comments, taking into account their sentiments, upvotes, and linguistic range. It allows the researchers to comprehensively understand the evolution of discussions, the quality of interactions, and the range of issues discussed. This approach aligns with user-centred design principles and digital ethnography, highlighting the importance of user-generated material in understanding digital behaviours and interactions. The dataset helps address a research gap in online debates by offering a specific, data-driven basis for examining the complexities of digital conversation dynamics.

## 3 Data Description

This dataset is a compilation of end-user discussions from Reddit, explicitly focusing on various features and updates related to the Google Maps application. These discussions are captured across seven distinct topics, reflecting diverse user interactions and feedback. Below, we provide a detailed and comprehensive breakdown of the dataset's quantitative and qualitative attributes, investing a sense of relief and confidence in the audience about the findings.

**Quantitative Overview**:

• Total Comments: 3119
• Topics Covered: 7
• Unique Authors: 1979
• No. of feature annotated: 722
• No. of issues identified from end-user feedback: 348
• No of claims annotated in the dataset: 1974
• The claims annotated in the dataset are further classified as supporting arguments, attacking arguments, and neutral as 702, 563, and 711, respectively.

**Qualitative Overview**:

• The dataset annotated raw end-user discussion from the Reddit forum into useful and requirements-related information, which software developers and vendors can use for improving the existing quality of the software application by providing remedies to the frequently reported issues and incorporating new features that are conflict-free and is frequently asked for.
• Additionally, the dataset provides an argumentative structure, i.e., positive, negative, and neutral arguments that can be used for informed decision-making underneath rationale, making the dataset unique from other requirements-related datasets that only comprise new features, issues, non-functional requirements, etc.
• The argumentative form of end-user comments provides prosperous qualitative data, offering insights into user sentiments and priorities.

A few instances from the dataset are shown in Table 2, which has been curated by collected end-user discussion across seven different discussion topics related to the FM application on the Reddit forum, as shown in Table 2. The main discussion topics and many end-user comments submitted and collected against them are listed in Table 3. Moreover, a detailed explanation of various attributes the proposed dataset contains is explained below to provide a better overview to potential readers, as shown in Table 2:

**Table 2:** Summary of the dataset.

S/No.	Discussion topic	No of comments
1	Google Maps is testing a combined commute to replace driving and transit	https://www.reddit.com/r/Android/comments/9clgh7/
2	Google Maps introduces location sharing	https://www.reddit.com/r/Android/comments/60v5za/
3	Take control of your commute with Google Maps	https://www.reddit.com/r/Android/comments/9kfdyl/
4	I wish Google Maps had an option to give me less verbose directions, but not entirely mute	https://www.reddit.com/r/Android/comments/4bxtcm/
5	Google Maps will soon allow you to share your ETA, add a shortcut to routine destinations, and create a map of your location history	https://www.reddit.com/r/Android/comments/4lpvk6/
6	Google Maps now warns you if you are navigating to a place that will be closed when you arrive	https://www.reddit.com/r/Android/comments/39yslr/
7	Google Maps for Android is finally rolling out multi-waypoint directions	https://www.reddit.com/r/Android/comments/4qg62x/
	**Total Reviews extracted**	**3119**

**Table 3:** Labeled Dataset and attributes with examples.

comment_Text	comment_ id	comment_ upvotes	parent_ id	Rationale_ Type	Claim_ Type	Have_ Rationale
People still do racial checkbox ads?	dfadazi	−1	60v5za	claim	neu	no
This is really cool for people who have friends.	df9vhad	−1	60v5za	claim	supporting	yes
Anyone else find it weird they were using Nexus 6p (or the similar looking huawei device) instead of pixels? Wonder if googles stocks are so low...	dfav8lp	0	60v5za	issue		yes
THIS FEATURE NEEDS TO BE IMPLEMENTED INTO ANDROID AUTO!	dfafofy	0	60v5za	feature		yes
I can see this being abused in manipulative relationships. I'm not saying it shouldn't exist, but I can see this raising complex ethical dilemmas.	dfa9gy3	0	60v5za	claim	attacking	yes

**Column 1—Comment Text (comment_text):** This column includes the end-user's comment text, offering direct insight into what users discuss and share about the GM app**.**

**Column 2—Comments Author (comment_author):** This column provides the username of the person who submitted feedback in the Reddit forum, shedding light on the various participants in the conversation.

**Column 3—Comment ID (comment_id**): This column represents a unique identifier for each end-user comment submitted in the Reddit forum, which we used to identify the argumentative structure of the comments.

**Column 4—Comment Upvotes (comment_upvotes):** This column indicates the “karma score” provided by the Reddit forum for each submitted comment, resulting from upvotes and downvotes by other registered users in the Reddit forum.

**Column 5—Parent ID (parent_id):** This column shows the parent ID of each end-user comment, identifying whether it's a direct reply to the original post (using a submission ID) or a response to another comment. Parent ID and Comment ID are used to identify the argumentative structure between the comments, i.e., which comments come after which comment in the discussion.

**Column 6—Rationale Type (rationale_type):** This column is user-generated and shows the requirements-related information associated with each end-user feedback. It could include whether the comment represents a claim, elaborates on an issue, or suggests a new feature. This field is utilized to identify whether the proposed new feature or issue reported is conflict-free or not using the theory of argumentation. Also, various machine and deep learning algorithms are used to classify end-user feedback from the Reddit forum into new features, issues, or claims. If the claim type is identified as the claim, its sub-type can be identified as supportive, neutral, or attacking in the rationale Type, elaborated below.

**Column 7—Claim Type (claim_type):** Similarly, this column is user-generated and shows the rationale information associated with the end-user feedback collected from the Reddit forum. The annotators assigned the value of supporting, neutral, or attacking to the end-user comment if the rationale type is identified as “Claim.” This field is utilized to identify whether the identified new feature or issue is conflict-free or not using the theory of argumentation. Also, it is used to train machine or deep learning classifiers to identify the claim type if its rationale type is “Claim.”

**Column 8—Have Rational (Have_Rational):** Finally, this column shows whether the end-user comment contains rational information or not. This field is utilized to train the classifiers to filter out end-user feedback that does not contain useful information for software evolution.

## 4 Study Design, Materials, and Methods

For Market-based software applications, end-users use various social media platforms to record their voices for continuous improvements or register their complaints for frequently occurring issues [10]. Therefore, software vendors need to listen to these voices and incorporate them into the software evaluation process to better user satisfaction. Although platforms like Reddit forum are less traditionally utilized for gathering software feedback compared to App stores, Amazon reviews, and Twitter, they offer unique advantages where developers can record rationale while making informed-decision. In our approach, we detailed the process of identifying and extracting software-related information from Reddit forum as an alternative source for CrowdRE. This platform provides a rich, alternative source for insights into software evolution, offering in-depth discussions and user engagements that are valuable for informed decision-making. For this purpose, we developed a Python script to extract software-related information from the Reddit forum while keeping the original structure of user discussion in the forum. The pseudo-code for extracting user conversation about software applications is elaborated in the paper. With this approach, we extracted 3119 end-user conversations on GM apps from seven related topics. Moreover, to enhance the readability and suitability for the software researchers and vendors, the data is annotated for further analysis of software evolution and improvements. This results in the development of a holistic CSV file that is compatible with various programming platforms, improving itss use and analysis. Below, we elaborate on the steps in regenerating and extracting end-user discussion from the Reddit forum using the provided code script.

1. To extract user conversations about the GM app from the Reddit forum, we first need to import Python PRAW API, Pandas for data organization, and DateTime for managing temporal data.
2. To regenerate and extract end-user conversations from the Reddit forum, you need to obtain a unique Client ID and secret key with a registered email. Once used, the key cannot be reused for another system.
3. Next, to extract the end-user conversation from the Reddit forum, one needs to specify the unique ID or title of the discussion topic, as shown in the code script. For example, in the code script, putting “9clgh7” or “Google Maps is testing a combined Commute tab to replace Driving and Transit” will extract the end-user conversations registered by end-users against this discussion topic in the same format as shown in the forum.
4. A Python function is created to extract the required data from the Reddit forum for the particular discussion topic. The function also checks if the end-user comment contains replies, which should be extracted as well.
5. Also, the user comments extracted from the Reddit forum are organized as they are presented in the Reddit forum. For example, the code script extracts the main discussion topic and all the replies to it. Similarly, if the reply comment receives another reply, it extracts that up to n levels.
6. With each extracted end-user comment, the author name, karma score, comment ID, and its related parent ID are extracted, as shown in Table 3.
7. Finally, the end-user discussion against the main discussion topic is converted to a CSV file while preserving the original discussion structure, i.e., the comment-reply for easy use and analysis.

To make the dataset pursuable for the machine and deep learning classifiers, we followed a thorough annotation process using grounded theory and a content analysis approach. The annotation process has been explained in detail in previously published approaches, i.e., CrowdRE-Arg [3] and CrowdRE-Varg [2]. In summary, we followed the process by first manually analyzing the end-user feedback on the Reddit forum to identify frequently occurring requirements-related concepts. We identified that end-users report frequently occurring issues, suggest new features, and register claims for the proposed new features or issues in an argumentative structure (comment-reply). The end-user's claims can further be divided into supporting, attacking, and neutral claims. Next, we developed a novel coding guideline using a grounded theory approach that contains definitions of new features, issues, and claims(supporting, attacking, and neutral) with examples.

The end-user feedback in the dataset is then annotated with the identified requirements-related elements using content analysis and developed coding guidelines. For this purpose, an Excel document containing all the end-user comments from the discussion topics and coding guidelines is provided to the two coders, who are experts in software engineering. Each coder individually annotates end-user feedback in the dataset by analyzing each review collected from the Reddit forum. Upon completing the individual task, the annotation from both coders is combined to identify the Inter-Annotator Agreement and Cohen's Kappa. The average inter-coder agreement between the two coders was 90 %, while Cohen's kappa for the two coders was 67 %, which is considered a substantial agreement on Cohen's kappa scale. Moreover, the annotation process was conducted iteratively, and possible disagreements between the annotators were resolved through continuous negotiation and discussions.

Code Snippet: The subsequent code snippet extracts end-user discussion from the Reddit forum while keeping the structure (comment-reply) of user discussion intact.

**Code Snippet:** The subsequent code snippet extracts end-user discussion from the Reddit forum while keeping the structure (comment-reply) of user discussion intact.

## Limitations

One limitation of the proposed approach is that the end-user conversation extracted from the Reddit forum is limited to the GM app only, which is the most discussed topic on the Reddit forum related to extracting software-related information underneath rationale. Also, the size of end-user comments in the dataset is limited to 3119, which is comparatively less than the dataset extracted from app stores and Twitter (X). However, the purpose is to conduct an exploratory and experimental study on alternative sources such as Reddit forums, where end-users have unlimited rounds of discussion on certain issues or new features related to software applications, unlike app stores and Twitter (X). Moreover, the end-user comments extracted from the Reddit forum are annotated by human coders to propose automated approaches, which might pose the threat of having a second guess on identifying the requirements-related label. Also, the code script is limited to self-cleaning or removing the end-user conversation whose root comment is deleted either by the users or by the forum administrator. Therefore, the user would have to manually clean the dataset by deleting the end-user comments whose root feedback is deleted, i.e., the comment against the fellow comments is registered. Moreover, the end-user comments in the dataset might not match the user conversation to be extracted for the same discussion topics in the Reddit forum. The reason is some comments are either deleted by the users or forum administrators. The code script provided is tested and extracting information from the Reddit forum, but the number of comments may vary.

## Ethics Statement

The study does not involve any animal or human subjects and does not collect direct opinions from the users using predefined surveys or questionnaires. Therefore, it did not require any ethical approval. Moreover, we confirm that a) participant data has been fully anonymized, and b) the Reddit forum data redistribution policies were complied with.

## CRediT authorship contribution statement

**Javed Ali Khan:** Methodology, Conceptualization, Writing – original draft, Writing – review & editing. **Nek Dil Khan:** Methodology, Writing – original draft. **Muhammad Yaqoob:** Software, Data curation, Investigation, Writing – review & editing. **Affan Yasin:** Software, Validation, Writing – review & editing. **Ayed Alwadain:** Methodology, Funding acquisition, Writing – review & editing.

## Data Availability

mendeleyUser discussions about Google Maps apps on the Reddit Forum: A Crowd Requirements Dataset (Original data).
